# Parental gonadossomatic mosaicism in *HIVEP2*-related intellectual disability and impact on genetic counseling–case report

**DOI:** 10.3389/fgene.2023.1156847

**Published:** 2023-06-27

**Authors:** Maria Abreu, Tiago Branco, Sónia Figueiroa, Cláudia Falcão Reis

**Affiliations:** ^1^ Medical Genetics Department, Centro de Genética Médica Jacinto de Magalhães, Centro Hospitalar Universitário do Porto, Porto, Portugal; ^2^ Pediatric Department, Centro Hospitalar Tâmega e Sousa, Penafiel, Portugal; ^3^ Division of Pediatric Neurology, Department of Child and Adolescent, Centro Hospitalar Universitário do Porto, Porto, Portugal; ^4^ Life and Health Sciences Research Institute (ICVS), School of Medicine, University of Minho, Braga, Portugal; ^5^ICVS/3B’s—PT Government Associate Laboratory, Braga/Guimaraes, Portugal

**Keywords:** intellectual development disorder, *HIVEP2*, MRD43, genetic counselling, case report

## Abstract

Intellectual development disorder, autosomal dominant 43 (MRD43) is an autosomal dominant disorder caused by heterozygous mutations in the *HIVEP2* gene. In this report, we describe a case of a 4-year-old boy with global development delay, hypotonia, and dysmorphic features, in whom the finding of a heterozygous nonsense pathogenic variant in exon 5 of *HIVEP2* [c.2827C>T p. (Arg943*)] through WES established a MRD43 diagnosis. Our patient’s phenotype overlaps with other MRD43 descriptions in the literature. Unlike previously reported cases, where the condition was almost invariably *de novo*, the healthy mother in this case presented mosaicism for the pathogenic variant. Thus, the recurrence risk increased significantly from 1% to up to 50%. The description of a variant inherited for MDR43 is singular in the literature and this description highlights the importance of parental studies for accurate genetic counseling, particularly for family planning.

## Introduction

Moderate to severe intellectual disability with autosomal dominant inheritance (MRD) is very often caused by *de novo* variants (Steinfeld et al., 2016; Jain and Atwal, 2019). Since this is often presumed to be the case for pathogenic variants in conditions with complete penetrance, segregation studies are seldom performed. In those instances, a theoretical recurrence risk estimated at 1% or lower is given to healthy parents due to the possibility of gonadal mosaicism.

MRD 43 (OMIM #616977) is an autosomal dominant disorder caused by heterozygous deleterious variants in the *HIVEP2* gene (Park et al., 2019).

*HIVEP2* (OMIM *143054) is located on chromosome 6q23-q24 and encodes the human immunodeficiency virus type 1 enhancer binding protein 2 involved in brain development. It regulates the somatostatin receptor (SSTR-2), c–myc, and genes of the NF-kB pathway (Srivastava et al., 2016; Steinfeld et al., 2016; Goldsmith et al., 2019). *HIVEP2* activates SSTR-2 transcription, and both are expressed in the frontal cortex and hippocampus (Rauch et al., 2012). C-myc is a transcription factor that regulates cell growth, differentiation, and apoptosis. In turn, the genes of the NF-kB pathway participate in neuronal development (Steinfeld et al., 2016).

*HIVEP2* also plays a key role in cellular immunity, adipogenesis, and bone remodeling (Park et al., 2019).

Patients with *de novo* loss-of-function variants in *HIVEP2* have neurodevelopmental disorders characterized by developmental delays, intellectual impairment, hypotonia, and mild dysmorphic features. Behavioral issues have also been reported, including autism spectrum, anxiety, oppositional defiance, hyperactivity, and attention deficit disorders (Srivastava et al., 2016; Jain and Atwal, 2019).

The condition was first associated with the phenotype by Raunch et al., in 2012 (Rauch et al., 2012), identified *de novo* in a patient from a developmental disorder network, through trio whole exome sequencing. It has since been described in at least 17 additional patients with variable features (comparison can be found in the supplementary files).

With one exception (Mo et al., 2022), all previously reported cases have documented the condition as being *de novo*, suggesting a low recurrence risk for future siblings of an affected individual. In our report, the variant was inherited from a healthy parent, which highlights the importance of parental studies for accurate genetic counseling regarding this condition.

## Materials and methods

The etiological investigation was performed in a clinical setting. Informed consent for publishing was obtained from the patient’s family. A review of the literature was performed by searching for the terms “MRD43” and “*HIVEP2*” in the Pubmed database.

## Case report

A 33 months male presented to genetic outpatient care and was referred from Neuropediatrics consultation due to a development delay of unknown etiology in a family seeking genetic counseling. He was the first child of a non-consanguineous, healthy Portuguese couple, with unremarkable family history.

First-trimester ultrasound showed an increased fetal nuchal translucency (percentile >99). The chorionic villi sample karyotype was 46,XY. Decreased fetal movements prompted delivery by cesarean section at 39^+3^ week gestational age. The APGAR scores at the first/fifth/10th minutes were 9/10/10. The birth weight was 3,090 g (percentile 21.4), the length was 48 cm (percentile 12.3), and the head circumference was 34 cm (percentile 30.2). The neonatal period was uneventful. Development was delayed: acquired cephalic control at approximately 4 months, unsupported sitting at 9 months, shuffling at 18 months, unassisted gait at 21 months, and first words at 24 months. At 33 months, evaluation using the Griffith’s Mental Development scale showed a GDQ of 51, compatible with a mild/moderate global development delay.

He had mild facial dysmorphic features: hypertelorism, epicanthus, telecanthus, a depressed nasal bridge and a wide nasal base, a wide cupid bow and a pointy chin. Tapering fingers and fifth finger clinodactyly were also noted. Physical examination was otherwise unremarkable.

From the investigations carried out at 21 months, we highlight hyperphosphatemia (1,921 U/L), mild hyperammonemia (52.9 μmol/L), and mildly increased ASAT (41 U/L) and LDH (317 U/L). These findings were not confirmed upon later repetition. CDT and TIF were normal. Brain MRI showed moderately reduced encephalic volume and hypoplastic corpus callosum.

Genetic studies were made available in a clinical setting: genetic testing for fragile X syndrome was normal. Array-CGH (Affymetrix CytoScan in a peripheral blood sample) revealed no pathogenic findings. WES-based NGS panel (Illumina/Human All Exon V6, Agilent Technologies in a peripheral blood sample), with 1,502 genes related to developmental delays/intellectual disability, detected a recurring heterozygous nonsense pathogenic *HIVEP2* variant [c.2827C>T p. (Arg943 *)], establishing a diagnosis of MRD43 in our patient. Variant classification was performed using the ACMG criteria (Richards et al., 2015).

Parental studies by Sanger sequencing detected the variant in the mother in 15% of peripheral blood cells, simultaneously confirming the variant using a second methodology. The mother showed no signs of illness, had good academic performance with higher education, and her neurological examination was unremarkable.

The biochemical tests were repeated at 3 yo, with no abnormal findings. The diagnosis motivated an EEG, which was unremarkable. Griffith’s Mental Development scale, performed at 4 yo, reaffirmed global developmental delay, with worse performance in the practical reasoning subscale (QA, 63; QB, 50; QC, 52; QD, 47; QE, 44). At the age of 4, the child spoke approximately 20 words but phrase construction was yet to be acquired. Currently, anthropometry has progressed to tall stature (percentile >99), weight is evolving in the 95th percentile, and OFC in the 38th percentile.

There is no specific treatment for MRD43. However, follow-up was adjusted to the phenotype, and the child currently benefits from speech therapy, occupational therapy, psychomotricity, physiotherapy, and hippotherapy. The child had already been referred for early intervention. Follow-up is since maintained in Neurology, Child Psychiatry, and Pediatric Development consultations. A timeline of the events may be found in the supplementary materials.

The parents were concerned with the condition and wished to avoid recurrence in future siblings; therefore, reproductive options were made available to the couple, including invasive prenatal diagnosis through amniocentesis or chorionic villi biopsy, preimplantation genetic testing, and other alternatives (such as oocyte donation). Preimplantation genetic diagnosis (PGD) was the chosen option, which would not have been considered in the absence of parental mosaicism. The recurrence risk for other family members was considered comparable to the general population.

## Discussion

MDR 43 is an autosomal dominant disorder caused by deleterious *HIVEP2* variants. Its phenotype is non-specific, and diagnosis of cases in the literature is generally obtained through whole exome studies, as in our patient. The physiological role of *HIVEP2* in development is still largely unexplained but has been purported to be related to its role as a transcription factor in the central nervous system and as an inflammatory mediator in the brain (Takagi et al., 2006; Zhao et al., 2019).

With the exception of patient 6 in the cohort of Mo et al., in 2022 (Berryer et al., 2013; Goldsmith et al., 2019; Jain and Atwal, 2019; Martínez-Glez et al., 2020; Mo et al., 2022; Park et al., 2019; Rauch et al., 2012; Richards et al., 2015; Shieh et al., 2020; Srivastava et al., 2016; Steinfeld et al., 2016; Takagi et al., 2006; Zhao et al., 2019), all previously described cases in which parental studies had been performed reported the variants to be *de novo* (Mo et al., 2022; 1-14). This patient’s family had self-reported that the variant was inherited from a healthy parent; however, details on possible mosaicism for the variant and parental evaluation are unavailable.

To the best of our knowledge, there is no international consensus on the recommendation of segregation studies on healthy parents for pathogenic variants with complete penetrance. However, given the phenotype’s severity and the parent’s planning on a future pregnancy, parental studies were conducted. The heterozygous pathogenic variant in exon 5 of the *HIVEP2* gene [c.2827C>T p. (Arg943 *)] was found in 15% of the mother’s peripheral blood cells. This result established the presence of gonadosomatic mosaicism (A3, according to the six-attribute classification (Martínez-Glez et al., 2020)) for the MRD43-causing variant in the mother. We emphasize the fact that the mother had no phenotypical features suggestive of the condition or of a mosaic state.

Genetic counseling was adjusted to provide a recurrence risk of up to 50%, *in lieu* of the *a priori* theoretical recurrence risk of 1% or less. This drastic change in genetic counseling significantly impacted the reproductive options the parents considered, and the couple opted for preimplantation genetic diagnosis (PGD), which would not have been considered otherwise.

The maternal mosaicism was well tolerated, despite a low-to-moderate degree mosaic in the blood sample. In addition to the case reported by Mo et al., and considering that the authors were made aware of a second case of recurrence of the disorder through a patient’s association (which suggests gonadal mosaicism), parental mosaicism should always be considered during genetic counseling for MRD43. The recurrence risk for children of other family members on the maternal branch is low. Mosaics occur *de novo* on the carrier individual, and revertant mosaicism has not been described for MRD conditions.

The occurrence of gonadosomatic mosaicism in healthy parents is presumed to be rare but may be underestimated considering the increasing number of reports in other MRD-like conditions (Berryer et al., 2013; Shieh et al., 2020). Thus, it is our opinion that genetic testing for the causal variant should always be offered to parents, especially those considering further family planning. We also encourage further reports on mosaic cases of MRD conditions where this is rarely reported, and that future reviews concerning these disorders account for somatic and gonadal mosaicism in order to estimate the real prevalence of these cases.

## Data Availability

The datasets for this article are not publicly available due to concerns regarding participant/patient anonymity. Requests to access the datasets should be directed to the corresponding author.
