# Supplementary material for: Parental gonadossomatic mosaicism in HIVEP2-related intellectual disability and impact on genetic counseling–case report
Source: Front Genet. 2023 Jun 27;14:1156847. doi: 10.3389/fgene.2023.1156847 (PMC10333530; doi:10.3389/fgene.2023.1156847)
Supplement: Supplementary file 1 [file DataSheet2.PDF]

Table 1: Phenotype of patients in the literature

Table 1

	Our patient	Srivastava et al, 2016 - Patient 1	Srivastava et al, 2016 - Patient 2	Srivastava et al, 2016 - Patient 3 (also Raunch et al 2012)	Steinfeld et al, 2016 - Patient 1	Steinfeld et al, 2016 - Patient 2	Steinfeld et al, 2016 - Patient 3	Steinfeld et al, 2016 - Patient 4	Steinfeld et al, 2016 - Patient 5	Steinfeld et al, 2016 - Patient 6	Goldsmith et al, 2019 - Patient 1	Goldsmith et al, 2019 - Patient 2	Park et al, 2019 - Patient 1	Park et al, 2019 - Patient 2	Jain et al 2019	Gong et al 2021 - Patient 16
- Gender	M	F	M	F	F	F	M	M	F	M	M	F	F	F	M	M
- Age at the time of description (yo)	4 yo	4	3	21	7	14	10	2	11	6	10	8	27	23	5	7
- Variant (NM_006734.4)	c.2827C>T p.(Arg943 *)	c.2827C>T p. (Arg943*)	c.3556C>T p. (Gln1186*)	c.5737delG p. (Asp1913Metfs*15)	c.6475G>T p.(Gly2159*)	c.2857G>T p.(Glu953*)	c.5614dupG p.(Glu1872Glyfs*16)	c.1189G>T p.(Asp397Tyr)	c.6625dup p.(Tyr2209fs)	c.3434delC p.(Pro1145fs)	c.5935C > T p. (Arg1979*)	c.2956_2957del p. (Glu986Argfs*4)	c.6609_6616del, p. (Glu2204*)	c.6667C>T, p. (Arg2223*)	c.3742 C > T p. (Gln1248*)	c.5935C>T p. (Arg1979*)
- Inheritance	Inherited variant from mosaic mother	De novo	De novo	De novo	De novo	De novo	De novo	De novo	De novo	De novo	De novo	De novo	De novo	De novo	De novo	De novo
- Delayed psychomotor development / Intellectual disability	Yes (Mild/Moderate)	Yes (Moderate - severe)	Yes (Mild)	Yes (Moderate - severe)	Borderline	Yes	Yes	Yes	Yes (Mild)	Yes (Mild)	Yes (Moderate)	Yes (Severe)	Yes (Mild)	Yes (Mild)	Yes (Mild)	Yes
- Motor delay / clumsy gait	Yes	Yes	Yes	Yes	Yes	Yes	Yes	Yes	Yes	Yes	Yes	Yes	Yes	Yes	Yes	Yes
- Language delay	Yes	Yes	Yes	Yes	Yes	Yes	Yes	Yes	Yes	Yes	Yes	Yes	Yes	Yes	Yes	Yes
- Behavioral disorders	No	Yes	N/A	Yes	Yes	Yes	Yes	No	No	Yes	Yes	Yes	No	No	No	N/A
- Hypotonia	No	Mild	Moderate	Mild	Yes	Yes	No (Hypertonia)	Yes	Yes	No	Yes	Yes	Yes	No	No	Yes
- Microcephaly	No	No	No	Yes	Yes	Yes	Yes	No	No	No	Yes (borderline)	No	No	Yes	No	N/A
- Dysmorphic facial features	Epicanthus, telecanthus, Widely-spaced eyes, broad nasal root and wide , deppressed bridge, wide nasal base, pointy chin.	Widely-spaced eyes, broad nasal root, upturned nose, high arched palate, asymmetric crying facies	High forehead, medial eyebrow flare, widely spaced eyes, broad nasal root, small mouth, small chin	Minor synophrys, palpebral upslanting, small ears with attached earlobes, prominent nose, high nasal bridge, low columella, short filtrum	Mild retrognathia	Elongated and narrow face	Low anterior hairline, hirsute, prominent eyebrows, synophrys, epicanthal folds, mildly thickened helix & simple anti-helix in ears, mild dental crowding of the lower jaw	Small hands and feet	High nasal bridge, broad mouth, rather flat philtrum, mild bifrontal narrowing, mildly broad halluces, sacral dimple, broad thorax, mild finger webbing	Square face, high/ broad forehead, unilateral strabismus, high nasal bridge, columella under alae nasi, small square ears with transverse crease, small square teeth, microretrognat hia	Brachycephaly, broad forehead, widely-spaced eyes, wide mouth, thin upper lip, frontal upsweep	Low anterior hairline, long, narrow face, arched eyebrows, mild synophrys, widely set eyes, slightly downslanted palpebral fissures, broad nasal root, high nasal bridge, low set columella, small, widely spaced teeth, thin upper lip, micrognathia, squared superior portion of helix	Broad nose, narrow chin, thin upper lip	Long, narrow face, small ears	None described	Broad forehead
- Poor feeding	No	Yes	No	No	N/A	N/A	N/A	N/A	N/A	N/A	N/A	Yes	N/A	N/A	N/A	Yes
-Gastroesophageal reflux	No	No	No	No	Yes	Yes	Yes	No	No	No	No	No	N/A	N/A	N/A	N/A
- Constipation	No	No	No	Yes	N/A	N/A	N/A	N/A	N/A	N/A	No	No	No	Yes	N/A	N/A
- Tapering fingers	Yes	No	Yes	Yes	N/A	N/A	N/A	N/A	N/A	N/A			N/A	N/A	N/A	N/A
- Seizures	No	No	No	No	Yes	Yes	Yes	EEG abnormality	N/A	N/A	No	No	No	No	No	Yes
- Thin corpus callosum	Yes	Yes	Yes	No	No	No	No	No	No	No	No	No	N/A	N/A	N/A	No
- Cerebral atrophy	Yes	No	No	Yes	No	Yes	No	No	No	No	No	No	N/A	N/A	N/A	No
-Other features	Transient hyperphosphatemia	Unilateral hip dysplasia	Hypermeetropia	Gengival bleeding, high TSH, umbilical hérnia	Static encephalopathy, dystonia, fatigue	Strabismus, spasticity, cerebral pausa, quadriplegia, weakness, dystonia, anemia, nephrocalcinosis, hip dysplasia, syncope, dyspnea	Abnormal spetroscopy, milk protein intolerance, strabismus, spasticity, Parkinsonism, hemiparesis, recurring tonsillitis in childhood	Short stature, reactive airway disease	Incomplete myelination at 4 yo, hypermobility of finges	Amblyopia, strabismus, hypermetropia, bronvhial hyperreactivity	Hypertrichosis, hyperfagia	Short neck, pes planos, skoliosis		Upper airway infections, oligomenorrhea		Regression was described

	Miyamoto et al 2021 - Individual 5415	Quental et al, 2021 - Patient 1	Quental et al, 2021 - Patient 2	Mo et al, 2022 - Patient 1	Mo et al, 2022 - Patient 2	Mo et al, 2022 - Patient 3	Mo et al, 2022 - Patient 4	Mo et al, 2022 - Patient 5	Mo et al, 2022 - Patient 6	Mo et al, 2022 - Patient 7	Mo et al, 2022 - Patient 8	Mo et al, 2022 - Patient 9	Mo et al, 2022 - Patient 10	Mo et al, 2022 - Patient 11	Mo et al, 2022 - Patient 12	Mierlo et al 2023
- Gender	F	M	M	M	F	F	F	M	F	M	M	M	M	M	M	M
- Age at the time of description (yo)	4	10	11	6	10	6	11	6	4	5	11	6	6	3	12	22
- Variant (NM_006734.4)	c.2827C>T p.(Arg943*)	c.2827C>T p.(Arg943*)	c.6667C>T p.(Arg2223*)	c.1189G>T p.(Asp397Tyr)	c.2827C>T p.(Arg943*)	c.6964C>T p.(Gln2322*)	c.2905C>T p.(Gln969*)	c.5686C>T p.(Gln1896*)	c.5900delC p.(Ser1967Cysfs)	c.2827C>T p.(Arg943*)	c.5935C>T p.(Arg1979*)	c.3742C>T p.(Gln1248*)	c.6667C>T p.(Arg2223*)	c.5150dupA p.(Leu1718fs)	c.5935C>T p.(Arg1979*)	c.5863dup, p.(His1955fs)
- Inheritance	De novo	De novo	De novo	De novo	De novo	De novo	De novo	De novo	Inherited (unaffected parent)	De novo	N/A	De novo	De novo	De novo	N/A	N/A
- Delayed psychomotor development / Intellectual disability	Yes	Yes	Yes	Yes	Yes	Yes	Yes	Yes	Yes	Yes	Yes	N/A	Yes	Yes	N/A	Yes
- Motor delay / clumsy gait	Yes	Yes	Yes	Yes	Yes	No	No	Yes	Yes	No	No	N/A	No	No	N/A	N/A
- Language delay	Yes	Yes	Yes	Yes	No	No	No	No	Yes	Yes	Yes	N/A	Yes	Yes	N/A	N/A
- Behavioral disorders	No	No	Yes	Yes	Yes	No	Yes	Yes	No	No	Yes	N/A	No	Yes	N/A	Yes
- Hypotonia	No	Yes	No	Yes	Yes	Yes	Yes	Yes	Yes	Yes	Yes	N/A	Yes	No	N/A	N/A
- Microcephaly	No	No	No	Yes	No	No	No	Yes	No	No	Yes	N/A	No	No	N/A	N/A
- Dysmorphic facial features	None described	Frontal bossing, downslanting palpebral fissures	None described	N/A	N/A	N/A	N/A	N/A	N/A	N/A	N/A	N/A	N/A	N/A	N/A	N/A
- Poor feeding	N/A	No	Yes	Yes				Yes			Yes	N/A		Yes	N/A	N/A
-Gastroesophageal reflux	N/A	Yes	No	No	Yes	Yes	No	Yes	No	Yes	No	N/A	Yes	No	N/A	N/A
- Constipation	N/A	No	Yes	No	Yes	No	No	No	No	No	No	N/A	Yes	Yes	N/A	N/A
- Tapering fingers	N/A	N/A	N/A													N/A
- Seizures	No	No	No	Yes	No	No	Yes	Yes	No	No	No	No	No	No	No	N/A
- Thin corpus callosum	Yes	Yes	No	N/A	N/A	N/A	N/A	N/A	N/A	N/A	N/A	N/A	N/A	N/A	N/A	N/A
- Cerebral atrophy	N/A	Yes	No	N/A	N/A	N/A	N/A	N/A	N/A	N/A	N/A	N/A	N/A	N/A	N/A	N/A
-Other features		Cryptorchidism, alternativa estropia§		Hip dysplasia, asthma	Strabismus	Strabismus	asthma	Strabismus		Strabismus	Strabismus					An acute episode of catatonia, treated with ECT
