# Supplementary figures and images for: Parental gonadossomatic mosaicism in HIVEP2-related intellectual disability and impact on genetic counseling–case report
Source: Front Genet. 2023 Jun 27;14:1156847. doi: 10.3389/fgene.2023.1156847 (PMC10333530; doi:10.3389/fgene.2023.1156847)

Supplementary files:

Figure 1:

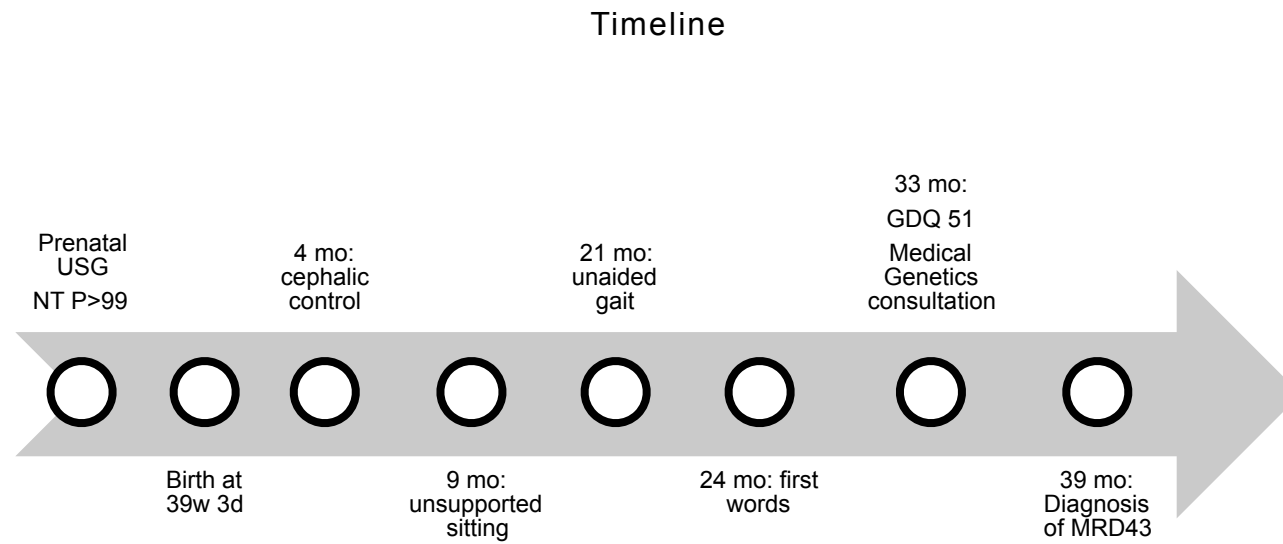

Supplement: Supplementary file 2 [file DataSheet1.PDF]
